# Latent tuberculosis infection screening and treatment outcomes in healthcare workers in Irish hospitals: a multi-centre cohort study

**DOI:** 10.1017/ice.2026.10439

**Published:** 2026-06

**Authors:** Melva Tan, Zainab Albaggal, Ciara Anderson, Daragh McGee, Cian Carey, Aoife Hehir, Dominick P. Natin, Maria Lenehan, Brian McCullagh, Lorraine Dolan, Eleanor Cronin, Noirin Noonan, Michelle Coleman, Anne Marie McLaughlin, Eileen Sykes, John Gallagher, Sarah O’Beirne, Deborah Moriarty, Grant Jeffrey, Eoin Feeney, Colm Bergin, Catherine Fleming, Arthur Jackson, Joseph Keane, Carlos Mejia-Chew, Liam Townsend

**Affiliations:** 1 Infectious Diseases, Mater Misericordiae University Hospital, Ireland; 2 Infectious Diseases, St Vincent’s University Hospital, Ireland; 3 Infectious Diseases, St James’s Hospital, Ireland; 4 Infectious Diseases, Galway University Hospital, Ireland; 5 Infectious Diseases, Mercy University Hospital, Ireland; 6 Occupational Medicine, Mater Misericordiae University Hospital, Ireland; 7 Respiratory Medicine, Mater Misericordiae University Hospital, Ireland; 8 Respiratory Medicine, St James’s Hospital, Ireland; 9 Respiratory Medicine, St Vincent’s University Hospital, Ireland; 10 Occupational Health, St James’s Hospital, Ireland; 11 Occupational Health, Mercy University Hospital, Ireland; 12 Workplace Health and Wellbeing Unit, Health Service Executive, Ireland; 13 Department of Medicine, University College Dublin, Dublin, Ireland; 14 Department of Clinical Medicine, Trinity College Dublin, Dublin, Ireland

## Abstract

**Objective:**

To evaluate factors associated with positive LTBI screening among HCWs and predictors of treatment initiation and completion across hospital sites in Ireland.

**Design:**

Multicentre retrospective cohort study.

**Setting:**

Five hospital sites in Ireland.

**Participants:**

N = 755 healthcare workers (HCWs).

**Methods:**

Evaluation of latent tuberculosis infection (LTBI) by interferon gamma release assay in HCWs from high-incidence countries during 2023, identified via occupational health records. IGRA positivity rates, linkage to treatment and treatment outcomes were recorded. Demographic and occupational factors associated with these outcomes were investigated.

**Results:**

There were n = 755 HCWs from high-incidence TB countries identified via occupational health records eligible for LTBI screening. 719 underwent IGRA testing, of whom 93 (13%) were positive. Age > 50 was associated with IGRA positivity (OR 5.71; 95% CI 1.79–18.17; *P* = .003). In addition to these n = 93 HCWs, two additional sites provided treatment outcomes for n = 164 HCWs, and a further n = 58 IGRA-positive HCWs were referred to Site 1. Among these 313 IGRA-positive HCWs, 50% initiated therapy, with substantial variation across sites (27%–88%). Multivariable analysis showed study site, but not demographic factors, predicted treatment initiation (*P* < .001). Common reasons for non-initiation included treatment refusal and non-attendance. Treatment completion was high (82%) and was not associated with study site.

**Conclusions:**

LTBI prevalence among HCWs in Ireland was lower than international estimates. While treatment initiation was low, completion was high. Treatment initiation varied by site, driven by institutional rather than individual factors. A standardised national programmatic approach is needed for HCWs within the LTBI cascade of care.

## Introduction

Tuberculosis (TB) remains a global public health concern, with an estimated 23% of the world’s population harbouring latent TB infection (LTBI).^1^ LTBI represents a critical reservoir for future active TB cases and is a central focus of the WHO End TB Strategy.^2^ Treatment of LTBI significantly reduces the risk of progression to active TB, especially in high-risk populations such as healthcare workers (HCWs), who are disproportionately exposed to TB due to occupational contact.^3,4^

TB activation in migration from countries with a high burden of TB to low-incidence settings is a major driver of TB cases in countries of low-incidence, particularly among HCWs born in areas of high TB endemicity.^5,6^ LTBI screening is an important element of preemployment occupational health assessments in all countries, regardless of national TB burden.^7^ While traditionally this screening was performed using a tuberculin skin test, this has largely been replaced with interferon gamma release assays (IGRAs). Screening allows for timely treatment of LTBI, preventing development of active infection, preserving HCW health, and limiting both community and nosocomial transmission.^8^ Preventing the development of active TB also has significant economic benefits, both in terms of direct drug and treatment costs as well as loss of earnings.^9–11^

In recognition of this occupational risk, the Irish Health Protection Surveillance Centre (HPSC) recommends LTBI screening for all newly-employed HCWs arriving from high-incidence countries, defined as those with an estimated annual TB incidence of ≥ 40 per 100,000 population, based on WHO criteria.^12^ In the event of a positive screening test, LTBI treatment is recommended once active TB infection has been outruled. This is in keeping with guidelines from the European Centre for Disease Prevention and Control.^13^ While national guidelines recommend screening and treatment, there is no standardised referral pathway for the management and treatment of LTBI-positive individuals in Ireland. Additionally, no service is in receipt of resources to provide care for HCWs with LTBI. Management decisions, including whether to offer therapy, choice of regimen, and monitoring, are determined locally and vary by institution, with local arrangements also determining which specialty sees these individuals, e.g., respiratory medicine, infectious diseases. Treatment uptake among HCWs is also variable and may be influenced by multiple factors including perceived risk, side effect concerns, and systemic barriers.^14^ This lack of a robust management pathway increases the risk of missed diagnoses, missed linkage to care, and missed treatment opportunities, all of which increase the risk of TB reactivation and its associated severe consequences.^15^

This study aimed to evaluate current LTBI screening and treatment practices and outcomes across tertiary hospitals in the Republic of Ireland over a 12-month period. We also aimed to identify factors associated with treatment initiation and completion for IGRA-positive HCWs.

## Methods

### Study design and data collection

Five tertiary hospitals in cities in Ireland (three in Dublin, one in Galway, and one in Cork) participated in this study. These centres all have on-site occupational health, respiratory, and infectious diseases services. Local policy at each hospital dictates which service assesses LTBI treatments. As such, each hospital is considered a study site, rather than the individual service. Sites were asked to provide data on TB screening for all newly-employed HCWs from a high-incidence country during a twelve-month period from January–December 2023. This only included HCWs who are newly arrived to Ireland, and do not include HCWs from a country of high-incidence moving from one healthcare centre to another within the country of Ireland, regardless of region of birth. Screening is not routinely performed on HCWs from low-incidence countries. All screens were performed using the QuantiFERON-TB Gold^©^ (Qiagen) enzyme immunoassay. Indeterminate or inconclusive samples on initial screen were repeated. Due to differences in reporting standards across sites, only a positive or negative result was reported, with negative control values, TB antigen values, and mitogen values not available.

Sites were also asked to provide data on linkage to care in the event of positive IGRA, as well as treatment outcomes. The minimum dataset required for a site to be included in the study was treatment outcome data, with no imputation of missing variables used. HCWs with positive IGRAs during this period were identified by accessing laboratory results and occupational health records, and onward referral, initiation, and completion of LTBI treatment were recorded. One study site accepted external referral for treatment consideration of HCWs with positive IGRAs, and these were included in the treatment analysis. HCWs with known positive IGRAs but documentation of prior LTBI treatment or prior TB infection were not included in the screening analysis. All eligible HCWs were included in the screening analysis. Demographic information collected included age, sex, and region of origin, as well as HCW role. Age was banded into deciles to prevent identification of individual HCWs. Similarly, country of birth is reported only if more than one HCW was included and was not broken down by site to avoid identification of individuals. HCW role was further dichotomised as clinical (patient-facing) and non-clinical (non-patient-facing). For HCWs with positive IGRAs, treatment initiation and completion were recorded, as well as reasons for not commencing or completing treatment. The reasons recorded for non-initiation were patient preference, decision to defer (a composite of patient and treating clinician decision), failure to attend appointments, and a history of prior TB/LTBI treatment. While on treatment monitoring varied across hospitals, all participants in receipt of LTBI treatment were required to attend monthly for surveillance bloods and prescription of monthly medication. Failure to attend monthly appointments resulted in treatment interruption and subsequent discontinuation. Treatment regimens were also recorded. We reported this study in line with the STROBE guidelines. (Supplemental Table 1).

Ethical approval for the study was granted by the St James’s Hospital Research and Innovation Office (Ref 2024-Nov-39893989) and the Mater Misericordiae University Hospital Ethics Committee (Ref 1/378/2554). Each participating site obtained local institutional approval for the secondary use of anonymised clinical data. As this was a retrospective service evaluation using de-identified data, formal individual informed consent was not required.

### Statistical analysis

Descriptive variables are reported as means with standard deviation (SD) or median with interquartile range (IQR), as appropriate. Univariate analysis for between-site and between-group differences was performed using chi-squared test (*χ* ^2^) for categorical variables, Wilcoxon ranked-sum (*z*) for continuous non-parametric variables, or ANOVA (*r* ^*2*^) for multiple categorical variables, as appropriate. Multivariable logistic regression was used to identify factors associated with positive IGRA, treatment initiation, and treatment completion. All analysis were performed using Stata version 18.0 (Stata Statistical Software, College Station, TX StataCorp LP) and statistical significance was indicated by *P* < .05.

## Results

**Figure 1.**
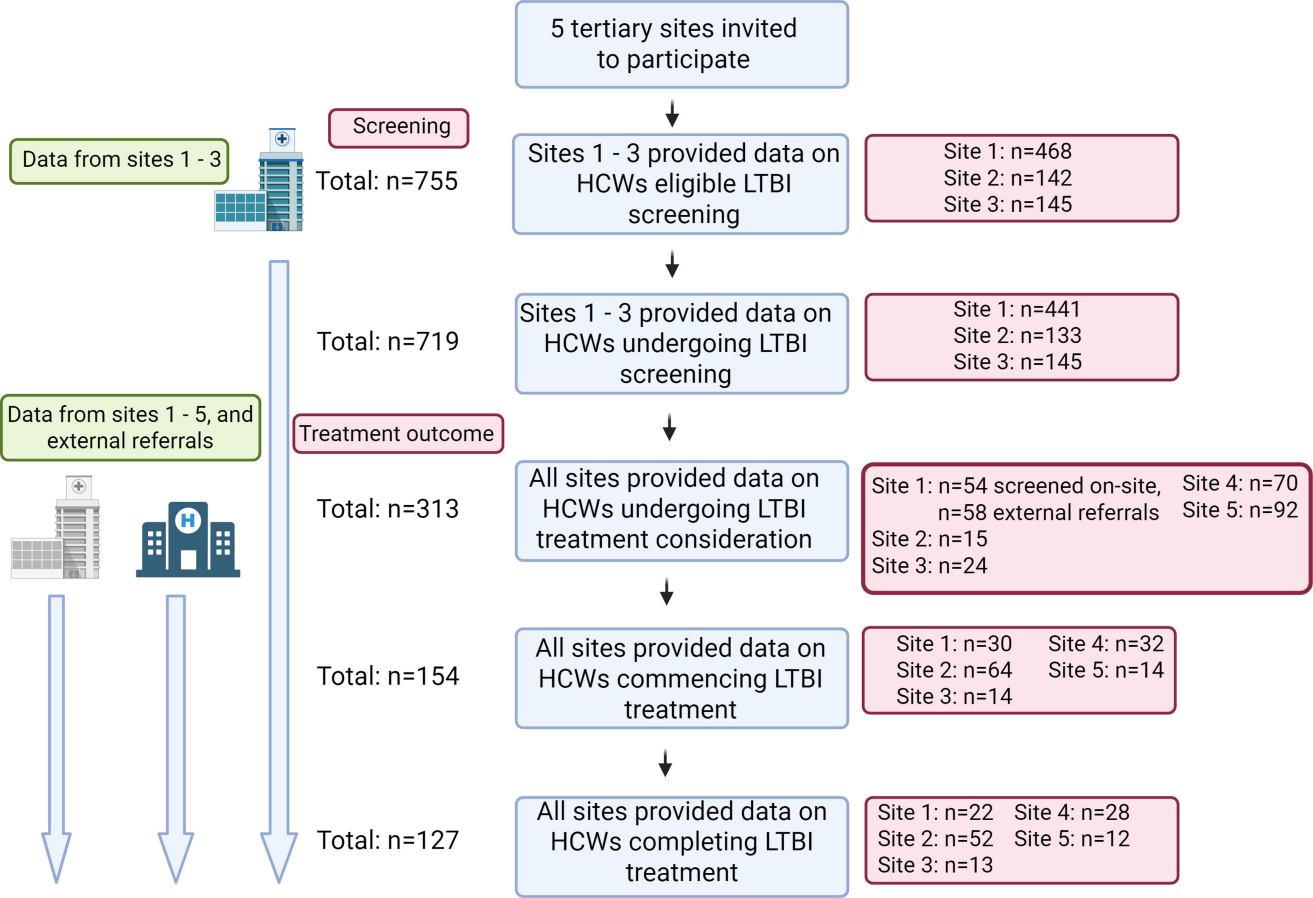
Figure 1 long description. Enrolment and data availability across sites. Site and participant information at each analysis step. Sites 1–3 had complete datasets including HCWs eligible for screening and treatment outcomes. Sites 4 and 5 had data only on treatment outcomes. Site 1 received n = 58 referrals for treatment consideration of HCWs with positive LTBI screens performed at external sites, and these were included in treatment analysis.

Five hospitals participated in this study, all of which were tertiary centres located in cities in Ireland. Of these, three sites could provide the full dataset of HCWs eligible for screening as well as treatment outcomes for IGRA-positive HCWs, while two sites could only provide data on treatment outcomes in HCWs with positive IGRAs. These latter two sites had incomplete data regarding the total eligible population for screening and could not be included in the screening analysis, but had complete records of all HCWs with positive IGRAs during the study period. The data collection process and site participation is shown in Figure 1.

There were n = 755 HCWs identified as eligible for LTBI screening across the three sites with available data. 95% (n = 719) underwent IGRA testing. Baseline demographics varied across the hospital sites (Supplemental Table 2). The majority of HCWs were from South and Central Asia and were nurses. The median age of the cohort was 33, and was predominantly female (80%). The overall IGRA positivity rate was 13% (n = 93), with no significant variation in positive IGRA rates across sites (*χ* ^2^ = 2.19, *P* = .33). Factors associated with IGRA positivity in this cohort were investigated. Having a positive IGRA was associated with being ≥ 50 years of age, but not associated with sex (Table 1). Coming from Sub-Saharan Africa was associated with positive IGRA (*P* = .04, *β* coefficient 0.08, 95% CI 0.01–0.16), while working as a healthcare attendant was also associated with positive IGRA (*P* = .04, *β* coefficient 0.09, 95% CI 0.01–0.18). These associations were further investigated using a multivariable logistic regression model, including the significant univariate factors (age, region of origin, and role). Age ≥ 50 remained a significant predictor of having a positive IGRA following adjustment (*P* = .003, OR 5.71, 95% CI 1.79–18.17).

**Table 1.** Cohort characteristics by interferon gamma release assay result Table 1 long description.

	Total cohort (n = 719)	IGRA negative (n = 626)	IGRA-positive, with % frequency of each characteristic (n = 93)	
Age, years;				*F* = 3.90, *r* ^2^ = .02,
<30	207	190	17 (8)	*P* = .01
30–39	376	324	52 (14)	
40–49	118	100	18 (15)	
≥50	18	12	6 (33)	
Sex,				*χ* ^2^ = .08, *P* = .77
Female; n	580	506	74 (13)	
Male; n	139	120	19 (16)	
Region of birth; n				*F* = 2.25, *r* ^2^ = .02,
South and Central Asia	428	372	56 (13)	*P* = .05
Sub-Saharan Africa Eastern Europe	83 26	65 23	18 (22) 3 (12)	
South America	16	15	1 (6)	
South East Asia	107	94	13 (12)	
Other	59	57	2 (3)	
Role; n				*F* = 2.16, *r* ^2^ = .01
Nurse	526	459	67 (13)	*P* = .06
Doctor	75	66	9 (12)	
Allied Health Professional	29	29	0 (0)	
Healthcare Assistant	64	50	14 (22)	
Laboratory	9	9	0 (0)	
Other	16	13	3 (19)	

ANOVA (*F* score, *r* ^2^) and Pearson’s Chi-squared test (*χ* ^2^) used, as appropriate. IGRA = interferon gamma release assay. Allied Health Professional is a composite of physiotherapists, occupational therapists, social workers, clinical nutritionists and speech and language therapists.

**Figure 2.**
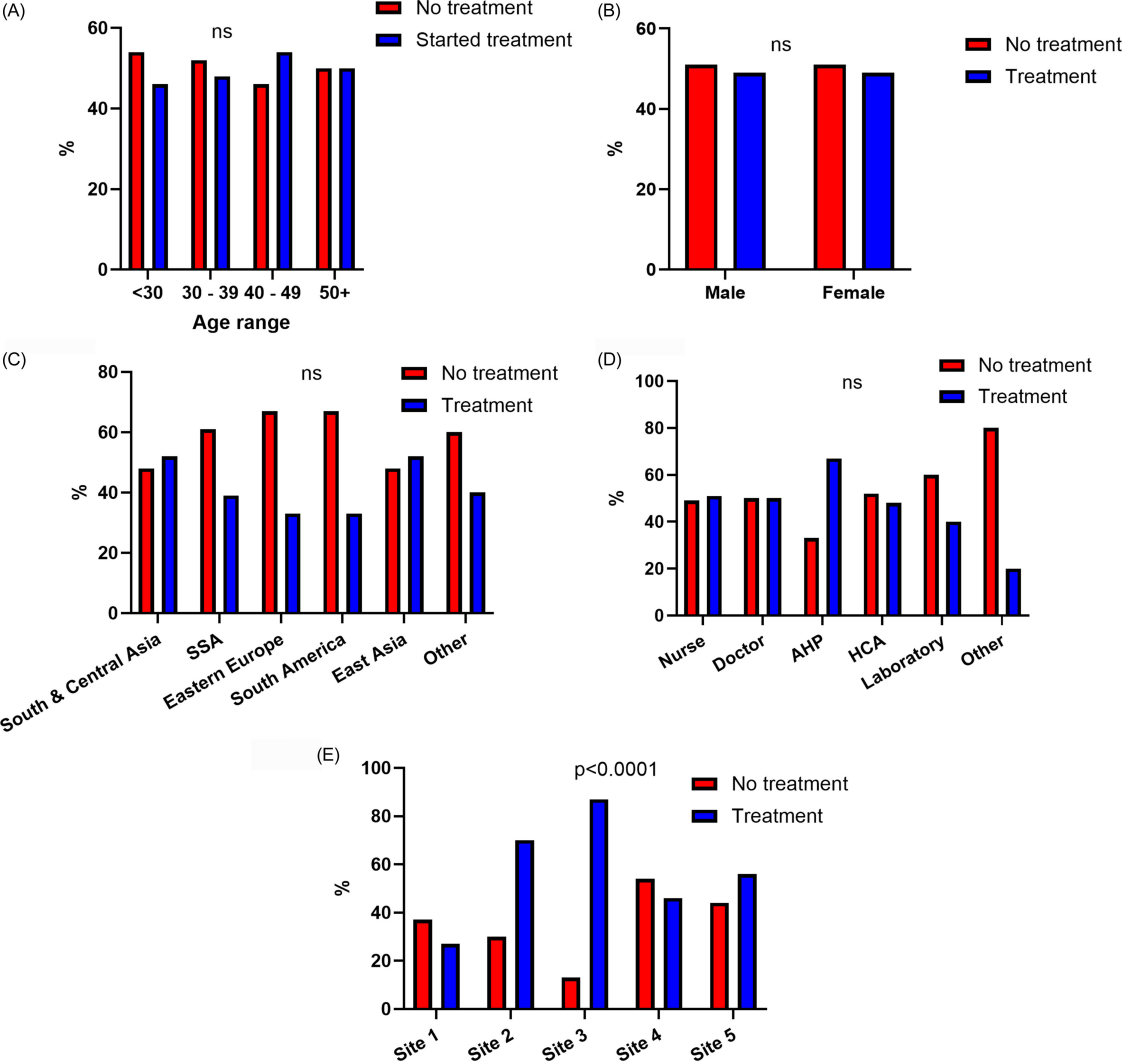
Figure 2 long description. Characteristics of HCWs commencing LTBI treatment. Comparing HCWs commencing LTBI treatment (blue) with those who did not (red) across (A) age, (B) sex, (C) region of birth, (D) HCW role, and (E) hospital site. N = 313 HCWs included in analysis. Differences assessed using ANOVA A, C, D and E and Pearson’s Chi-squared test for B. Percentages shown for each category. SSA = Sub-Saharan Africa; AHP = allied health provider; HCA = healthcare attendant. Allied health professional is a composite of physiotherapists, occupational therapists, social workers, clinical nutritionists and speech and language therapists.

The initiation and completion of treatment in HCWs from high-incidence countries with positive IGRAs were assessed. There were n = 313 HCWs with positive IGRAs across the five sites. This group was comprised of the n = 93 cohort identified via screening at the three sites with full screening data available, as well as an additional n = 164 HCWs with positive IGRAs from the two sites without available screening data. Furthermore, an additional n = 58 HCWs with positive IGRAs from external institutions were referred to Site 1 for treatment consideration. A complete breakdown is shown in Figure 1. All HCWs were informed of their positive IGRA results and offered appointments for treatment consideration. The median age of HCWs with positive IGRA was 35 years (IQR 8) and the cohort was predominantly female (n = 229, 69%). There was sociodemographic variation across the sites again noted, with variation in sex, region of birth, and staff role (Supplemental Table 3). The majority of HCWs with positive IGRA were nurses (72%), followed by doctors (10%) and healthcare assistants (15%). This is broadly reflective of the overall composition of patient-facing staff in the institutions included, with 17% of these being medical and 13% healthcare assistants. However, nursing are over-represented in the positive IGRA cohort, as they only represent 51% of patient-facing staff. While screening was conducted entirely by Occupational Health across all sites, HCWs with positive IGRAs were seen by Respiratory, Infectious Diseases, or Occupational Health services for treatment consideration. Half of the IGRA-positive cohort (154/313, 50%) initiated LTBI treatment. However, there was significant heterogeneity in commencing treatment across sites, with initiation rates ranging from 27%–88% (Supplemental Table 4). Factors associated with treatment initiation were evaluated. There were no sociodemographic characteristics associated with increased likelihood to commence LTBI treatment. However, study site attended was associated with the decision to commence therapy (Figure 2).

**Figure 3.**
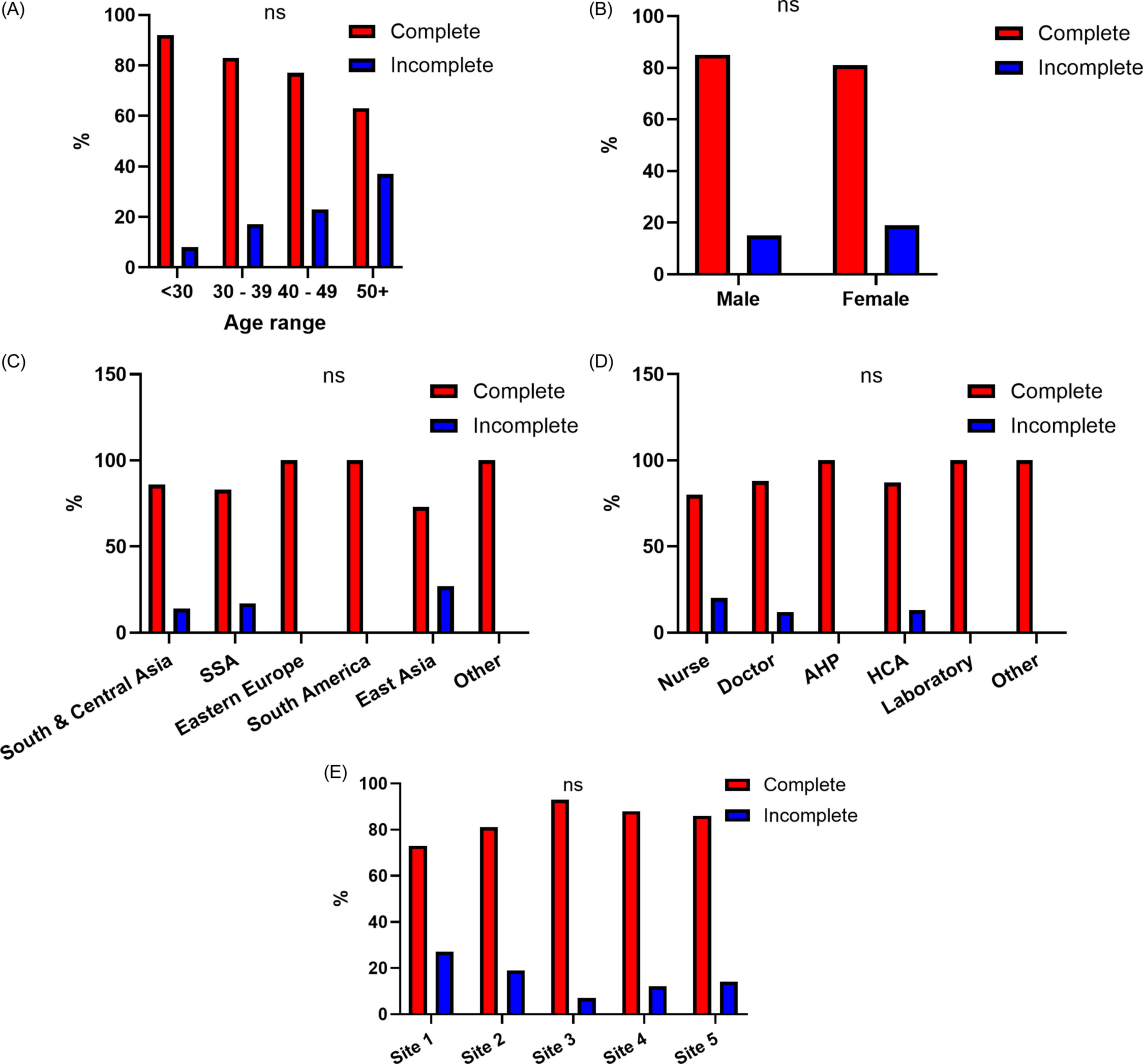
Figure 3 long description. Characteristics of HCWs completing LTBI treatment. Comparing HCWs completing LTBI treatment (red) with those who did not (blue) across (A) age, (B) sex, (C) region of birth, (D) HCW role, and (E) hospital site. N = 154 HCWs included in analysis. Percentages shown for each category. Differences assessed using ANOVA for A, C, D and E and Pearson’s Chi-squared test for B. SSA = Sub-Saharan Africa; AHP = allied health provider; HCA = healthcare attendant. Allied health professional is a composite of physiotherapists, occupational therapists, social workers, clinical nutritionists and speech and language therapists.

Treatment choice and outcomes were evaluated. Among those treated, 140/154 (91%) received rifampicin monotherapy for four months, with the remaining 14/154 (9%) receiving isoniazid monotherapy for nine months. Again, there was heterogeneity across sites, with rifampicin usage ranging from 43%–100% (Supplemental Table 4). Overall treatment completion was high at 82% (127/154), with no significant difference between centres. Factors associated with non-completion and the rationale for same were investigated. Failure to complete the treatment course was independent of any measured variable (Figure 3). The most common reason for non-completion was non-attendance to follow up appointments after treatment was commenced (11%), with adverse effects leading to treatment discontinuation in 8% of cases. There were no differences in treatment completion based on treatment choice with either rifampicin or isoniazid (*χ* ^2^ = .16, *P* = .69). Taken together, of the n = 313 HCWs with a positive IGRA, treatment completion rates were highly variable across sites, with 20% completion at site 1, 57% at site 2, 82% at site 3, 40% at site 4, and 24% at site 5. Influences on treatment initiation and completion were evaluated further through multivariate logistic regression, adjusting for age, sex, and region of birth. The study site remained significantly associated with commencing LTBI therapy. No variables predicting treatment completion were identified following multivariable adjustment (Table 2).

**Table 2.** Associations with treatment commencement and completion Table 2 long description.

	Commencing treatment		Completing treatment	
	OR (95% CI)	*P* value	OR (95% CI)	*P* value
Age	0.84 (0.60–1.18)	.32	1.47 (0.83–2.62)	.19
Sex, male	1.02 (0.59–1.77)	.95	0.66 (0.23–1.86)	.43
Region of origin	0.98 (0.86–1.10)	.70	1.20 (0.97–1.48)	.09
Study site				
- Site 1	Reference	n/a	Reference	n/a
- Site 2	0.16 (0.09–0.30)	<.0001	0.77 (0.26–2.24)	.63
- Site 3	0.05 (0.01–0.24)	<.0001	0.18 (0.02–1.75)	.14
- Site 4	0.44 (0.22–0.93)	.03	0.29 (0.06–1.33)	.11
- Site 5	0.29 (0.12–0.71)	.007	0.56 (0.10–3.30)	.52

N = 313 HCWs included in treatment initiation analysis; n = 154 HCWs included in treatment completion analysis. Multivariable logistic regression, with all variables shown included.

## Discussion

This multi-centre retrospective study found that LTBI screening was implemented in 95% of those eligible (719/755), and LTBI infection was found in 13% (93/719) of these HCWs. Only half of these commenced LTBI treatment. The study site attended by HCWs was identified as being most strongly associated with commencing LTBI therapy. For HCWs commencing treatment, we show high rates (82%) of treatment completion. We also demonstrate the logistical challenges facing LTBI screening in high-risk HCW populations within the healthcare service in the absence of a coordinated national service with incomplete datasets on HCWs eligible for screening, despite the setting of a resource-rich country. As a result, two of the five sites could not provide screening data and could only provide treatment outcomes on HCWs with positive IGRAs.

There is a large variation in IGRA positivity rates amongst HCWs reported across previous studies, which is unsurprising given the large geographical variation in the studies performed . Notably, older age has consistently been identified as a risk factor for IGRA positivity, in both high and low-incidence TB countries.^16^ Our results are consistent with previously published literature, with increasing age being the only variable associated with a positive IGRA result. While country and region of origin is an established risk factor for developing LTBI, we did not see any geographical differences here.^17^ This is unsurprising, as our inclusion criteria only included HCWs from high-risk countries in line with screening recommendations, lessening the effect of differing global disease burden.

Despite similar IGRA positivity rates across all sites included, there was significant inter-site variation in treatment initiation rates, ranging from 27 to 88% across hospitals. These differences were not explained by any recorded demographic or occupational factors, but seemed to be site-specific. This suggests that institutional practices or site-specific policies, as well as resource availability, may affect patient engagement and treatment decisions, even within a single jurisdiction. Heterogeneity in LTBI treatment initiation has previously been reported across other centres.^18^ Limiting the heterogeneity in the management of HCWs with LTBI through a programmatic approach could decrease dropouts and loss to follow up at all stages of the LTBI cascade of care.^19^ HCWs are particularly prone to poorer engagement with treatment at baseline compared to the general population.^20^ This emphasises the importance of minimising known variations that can occur with differences in treatment initiation based on the managing clinician.^21^ Directly observed therapy is regularly utilised in Ireland for patients with active TB infection. Utilising this in the setting of HCWs with LTBI may increase treatment adherence and completion.

Despite disparities in initiation, treatment completion was high (82%) among those who started therapy, which is comparable to that of the general population internationally.^22^ The predominant use of rifampicin monotherapy seen here is reflective of international guidance.^23^ Shorter treatment regimens are also preferred by patients and are associated with increased rates of completion.^24,25^ Nevertheless, we observed variation in treatment regimens, with isoniazid the drug of choice at one of the hospital sites. We did not identify any association between treatment choice and treatment completion. No patients received rifapentine, despite its utility in short course LTBI treatment.^26^ This may in part be due to challenges accessing this drug in Europe; it was not available in Ireland during the study period.^27^

More than half of IGRA-positive HCWs did not start treatment, with common barriers including treatment refusal and failure to attend appointments. Concerns about medication toxicity and the social stigma surrounding TB have been cited as barriers to LTBI treatment uptake, particularly among migrant populations.^28^ Qualitative research from low-incidence European settings has shown that stigma manifesting as fear of social exclusion, cultural shame, and discrimination including fears of deportation, can significantly discourage engagement with LTBI services among migrants.^29^ These obstacles highlight the need for enhanced and culturally-tailored patient education, effective counselling, and streamlined pathways to improve treatment uptake.

A significant finding from our study is the lack of data availability to allow comprehensive assessment of LTBI screening and treatment in an important cohort. Two of our study sites could not provide data on screening outcomes. In low-incidence countries such as Ireland, screening and treating LTBI in HCWs is strongly recommended.^30^ This not only protects HCW health, but has been shown to be cost-effective.^31^ A coordinated national approach, with clear guidance, referral pathways, and recording of treatment outcomes would improve engagement of both patients and providers, reduce variation in the care provided, and improve healthcare outcomes. The lack of a national referral pathway, centralised data system, or minimum dataset significantly hampers quality assurance, cohort tracking, and public health planning.

Strengths of this study include its multi-centre design, which allowed comparison of screening and treatment outcomes across diverse institutional settings. Centralised data collection enhanced the reliability and comparability of results. Inclusion of a large cohort of HCWs, a high-risk and strategically important group for TB control, further strengthens this study’s relevance. Detailed capture of demographic, occupational, and clinical variables allowed for robust analysis of factors influencing IGRA positivity and treatment outcomes, including multivariable modelling.

There are several notable limitations to this study. First, the data were not uniformly available across all centres leading to heterogenous datasets, and loss to follow up may have led to underestimation of true completion rates. This may have introduced ascertainment bias, while the use of only complete cases for analysis may reduce our statistical power. It demonstrates the need for robust documentation of treatment outcomes for all patients who embark on a treatment course, and not solely those who engage with care as per protocol. Additionally, the retrospective design introduces potential residual confounding from unmeasured variables. While all HCWs included in screening were undertaking their first HCW role in Ireland, data relating to how long they have been in the country prior to commencing employment or any non-healthcare employment history is not available. Additionally, prior known exposures to TB cases, either as part of occupation or within the HCW’s household, was not recorded. Therefore, it cannot be ascertained whether infection was acquired prior to or subsequent to the HCW’s arrival in Ireland. Furthermore, routine screening of HCWs from low-incidence countries is not performed, which prevents comparison with the rates seen in HCWs in this study. Reasons for treatment non-initiation or discontinuation were based on clinical documentation, were non-standardised. Although site-level differences were apparent, we did not assess specific institutional factors (e.g., staffing, appointment systems, or patient education materials) that might explain observed variability. Finally, only datasets from hospital sites were obtained. Findings may differ for HCWs in community-based healthcare facilities.

This study identifies LTBI in 13% of HCWs from high-risk regions migrating to an area of low TB endemicity, with increasing age being associated with positive LTBI screening test. Additionally, it demonstrates the heterogeneity in data collection, treatment initiation, and treatment choice that exists in the absence of a robust management framework, and advocates for the development of a national programme to manage LTBI.

## Supporting information

10.1017/ice.2026.10439.sm001 Tan et al. supplementary material Tan et al. supplementary material

## Supplementary material

The supplementary material for this article can be found at https://doi.org/10.1017/ice.2026.10439.

## Figure 1 Long description

The flowchart begins with the invitation of five tertiary sites to participate in the study. Sites 1 to 3 provided data on healthcare workers eligible for latent tuberculosis infection screening, with a total of 755 participants. The breakdown for these sites is 468 for Site 1, 142 for Site 2, and 145 for Site 3. Sites 1 to 3 also provided data on healthcare workers undergoing latent tuberculosis infection screening, totaling 719 participants, with 441 from Site 1, 133 from Site 2, and 145 from Site 3. All sites provided data on healthcare workers undergoing latent tuberculosis infection treatment consideration, totaling 313 participants. The breakdown includes 54 screened on-site and 58 external referrals for Site 1, 15 for Site 2, 24 for Site 3, 70 for Site 4, and 92 for Site 5. All sites also provided data on healthcare workers commencing latent tuberculosis infection treatment, totaling 154 participants, with 30 from Site 1, 64 from Site 2, 14 from Site 3, 32 from Site 4, and 14 from Site 5. Finally, all sites provided data on healthcare workers completing latent tuberculosis infection treatment, totaling 127 participants, with 22 from Site 1, 52 from Site 2, 13 from Site 3, 28 from Site 4, and 12 from Site 5.

Navigate back to Figure 1.

## Figure 2 Long description

The bar graph compares healthcare workers who commenced LTBI treatment (blue bars) with those who did not (red bars) across various categories. The x-axis represents different categories: age range, sex, region of birth, healthcare worker role, and hospital site. The y-axis represents the percentage of healthcare workers. The graph shows five grouped bar charts labeled A to E. Chart A compares age ranges: under 30, 30 to 39, 40 to 49, and 50 and above. Chart B compares sex: male and female. Chart C compares regions of birth: South and Central Asia, Sub-Saharan Africa, Eastern Europe, South America, East Asia, and other regions. Chart D compares healthcare worker roles: nurse, doctor, allied health provider, healthcare attendant, laboratory, and other roles. Chart E compares hospital sites: Site 1, Site 2, Site 3, Site 4, and Site 5. The data shows no significant differences in most categories except for hospital site, where Site 3 shows a significant difference with a p-value of less than 0.0001. All values are approximated.

Navigate back to Figure 2.

## Figure 3 Long description

The bar graph compares the percentage of healthcare workers (HCWs) who completed LTBI treatment (red bars) with those who did not (blue bars) across various categories. The x-axis represents different categories: (A) age range, (B) sex, (C) region of birth, (D) HCW role, and (E) hospital site. The y-axis shows the percentage of HCWs. In (A), age ranges are divided into <30, 30-39, 40-49, and 50+ years, with a higher percentage of younger HCWs completing treatment. In (B), both male and female HCWs show a higher completion rate. In (C), regions include South and Central Asia, Sub-Saharan Africa, Eastern Europe, South America, East Asia, and Other, with varying completion rates. In (D), roles include Nurse, Doctor, Allied Health Provider, Healthcare Attendant, Laboratory, and Other, with nurses and doctors showing higher completion rates. In (E), hospital sites are labeled as Site 1 to Site 5, with Site 3 showing the highest completion rate. The graph indicates that completion rates vary significantly across different categories. All values are approximated.

Navigate back to Figure 3.

## Table 1 Long description

The table presents data on cohort characteristics by interferon gamma release assay (IGRA) results. It includes columns for total cohort, IGRA negative, and IGRA-positive with percentage frequency of each characteristic. The table is divided into rows for age groups, sex, region of birth, and role. Age groups are categorized as under 30, 30-39, 40-49, and 50 or older. Sex is divided into female and male. Regions of birth include South and Central Asia, Sub-Saharan Africa, Eastern Europe, South America, South East Asia, and other. Roles include nurse, doctor, allied health professional, healthcare assistant, laboratory, and other. The table shows the number of individuals in each category and the percentage frequency of IGRA positivity. Notable trends include higher IGRA positivity rates in older age groups, particularly those 50 years and older, and among healthcare assistants. Additionally, individuals from Sub-Saharan Africa show a higher percentage of IGRA positivity compared to other regions.

Navigate back to Table 1.

## Table 2 Long description

The table presents data on factors associated with the commencement and completion of treatment. It includes columns for odds ratios with 95% confidence intervals and p-values for age, sex, region of origin, and study site. The table has five rows for different factors and two main sections for commencing and completing treatment. Notable trends include varying odds ratios for different study sites, with sites 2, 3, 4, and 5 showing significant differences in treatment commencement compared to the reference site 1. The p-values indicate the statistical significance of these associations.

Navigate back to Table 2.
