# Supplementary material for: Latent tuberculosis infection screening and treatment outcomes in healthcare workers in Irish hospitals: a multi-centre cohort study
Source: Infect Control Hosp Epidemiol. 2026 Apr 13;47(6):618–25. doi: 10.1017/ice.2026.10439 (PMC13216808; doi:10.1017/ice.2026.10439)
Supplement: Tan et al. supplementary material [file S0899823X26104395sup001.docx]

*Supplemental Table 1: STROBE checklist*

|  | Item No | Recommendation | Included | Relevant text from manuscript |
| --- | --- | --- | --- | --- |
| **Title and abstract** | 1 | (*a*) Indicate the study’s design with a commonly used term in the title or the abstract | Yes | Indicated in title |
|  |  | (*b*) Provide in the abstract an informative and balanced summary of what was done and what was found | Yes | Stated in abstract |
| Introduction | | |  |  |
| Background/rationale | 2 | Explain the scientific background and rationale for the investigation being reported | Yes | First three paragraphs of introduction |
| Objectives | 3 | State specific objectives, including any prespecified hypotheses | Yes | Final paragraph of introduction |
| Methods | | |  |  |
| Study design | 4 | Present key elements of study design early in the paper | Yes | First paragraph of methods |
| Setting | 5 | Describe the setting, locations, and relevant dates, including periods of recruitment, exposure, follow-up, and data collection | Yes | First and second paragraph of methods *Ten sites were invited to take part…* |
| Participants | 6 | (*a*) Give the eligibility criteria, and the sources and methods of selection of participants. Describe methods of follow-up | Yes | First and second paragraph of method  *Provide data on all TB screening…*  *Provide data on linkage to care…* |
| Variables | 7 | Clearly define all outcomes, exposures, predictors, potential confounders, and effect modifiers. Give diagnostic criteria, if applicable | Yes | Methods, paragraph 2. *Minimum dataset required ... was treatment outcome data*  *Demographic information …and HCW role* recorded. |
| Data sources/ measurement | 8 | For each variable of interest, give sources of data and details of methods of assessment (measurement). Describe comparability of assessment methods if there is more than one group | Yes | Second paragraph of methods. *…identified accessing laboratory results and occupational health records* |
| Bias | 9 | Describe any efforts to address potential sources of bias | Yes | Discussion. *…this may have introduced ascertainment bias … potential residual confounding from unmeasured variables* |
| Study size | 10 | Explain how the study size was arrived at | Yes | The inclusion criteria are outlined in the methods and visualised in Figure 1 |
| Quantitative variables | 11 | Explain how quantitative variables were handled in the analyses. If applicable, describe which groupings were chosen and why | Yes | Paragraph on *Statistical Analysis* in methods. Additional classification explained in methods section … *HCW role was further dichotomised…* |
| Statistical methods | 12 | (*a*) Describe all statistical methods, including those used to control for confounding | Yes | Paragraph on *Statistical Analysis* in methods. Univariate and multivariate models described. |
|  |  | (*b*) Describe any methods used to examine subgroups and interactions | Yes | VIFs were calculated for regression models to assess for collinearity |
|  |  | (*c*) Explain how missing data were addressed | Yes | This is addressed in the methods section: *No imputation of missing variables* |
|  |  | (*d*) If applicable, explain how loss to follow-up was addressed | Yes | Loss to follow up were included in the intention to treat analysis |
|  |  | (*e*) Describe any sensitivity analyses | N/A | Not applicable |

| Results | | | Included | Relevant text from manuscript |
| --- | --- | --- | --- | --- |
| Participants | 13 | (a) Report numbers of individuals at each stage of study—eg numbers potentially eligible, examined for eligibility, confirmed eligible, included in the study, completing follow-up, and analysed | Yes | Numbers included at each stage of results process, and in tables |
|  |  | (b) Give reasons for non-participation at each stage | Yes | Reasons for non-treatment included (Table 3) |
|  |  | (c) Consider use of a flow diagram | Yes | Provided in Figure 1 |
| Descriptive data | 14 | (a) Give characteristics of study participants (eg demographic, clinical, social) and information on exposures and potential confounders | Yes | Provided in results text and Table 1 |
|  |  | (b) Indicate number of participants with missing data for each variable of interest | Yes | Included in results text |
|  |  | (c) Summarise follow-up time (eg, average and total amount) | N/A | Not applicable |
| Outcome data | 15 | Report numbers of outcome events or summary measures over time | Yes | Treatment outcomes described in results text and Tables 3 &4, Figures 2 & 3 |
| Main results | 16 | (*a*) Give unadjusted estimates and, if applicable, confounder-adjusted estimates and their precision (eg, 95% confidence interval). Make clear which confounders were adjusted for and why they were included | Yes | Odds ratio and confidence intervals provided in Table 4, with legend describing variables included |
|  |  | (*b*) Report category boundaries when continuous variables were categorized | Yes | Median and IQR used for continuous variables |
| Other analyses | 17 | Report other analyses done—eg analyses of subgroups and interactions, and sensitivity analyses | Yes | Multivariable regression used to interrogate factors associated with positive IGRA (Table 2) and treatment outcomes (Table 4) |
| Discussion | | |  |  |
| Key results | 18 | Summarise key results with reference to study objectives | Yes | Throughout results section, in both the text and the Tables/Figures/Supplemental material |
| Limitations | 19 | Discuss limitations of the study, taking into account sources of potential bias or imprecision. Discuss both direction and magnitude of any potential bias | Yes | Paragraph in discussion, including comments on data availability, design limitations, heterogenous coding across sites, and limits on generalisability |
| Interpretation | 20 | Give a cautious overall interpretation of results considering objectives, limitations, multiplicity of analyses, results from similar studies, and other relevant evidence | Yes | Discussion of results includes references to previously published work, caveats to interpretation, and future directions |
| Generalisability | 21 | Discuss the generalisability (external validity) of the study results | Yes | Discussed in results section. *…this may limit the generalisability of our results…* |
| Other information | | |  |  |
| Funding | 22 | Give the source of funding and the role of the funders for the present study and, if applicable, for the original study on which the present article is based | Yes | No funding declaration necessary. Same stated in the Acknowledgements section. |

*Supplemental Table 2: Characteristics of healthcare workers undergoing latent tuberculosis infection screening by site*

|  | Total (n=755) | Site 1 (n=468) | Site 2 (n=142) | Site 3 (n=145) |  |
| --- | --- | --- | --- | --- | --- |
| IGRA performed, yes; n (%) | 719 (95) | 441 (94) | 133 (94) | 145 (100) | Χ^2^=9.06 p=0.01 |
| Positive IGRA, yes; n (%) | 93 (13) | 54 (12) | 15 (11) | 24 (17) | Χ^2^=2.19, p=0.33 |
| Age, years; n (%)   - <30 - 30 – 39 - 40 – 49 - ≥50 | 210 (28)  399 (53)  126 (17)  20 (3) | 128 (27)  233 (50)  90 (19)  17 (4) | 28 (20)  89 (63)  24 (17)  1 (<1) | 54 (37)  77 (53)  12 (8)  2 (1) | r^2^=0.02, p=0.001 |
| Sex, female; n (%) | 607 (80) | 382 (82) | 96 (68) | 129 (89) | Χ^2^=21.94, p<0.0001 |
| Region of birth; n (%)  South & Central Asia  Sub-Saharan Africa  Eastern Europe  South America  South East Asia  Other | 445 (59)  86 (11)  30 (4)  16 (2)  116 (15)  62 (8) | 262 (56)  58 (12)  26 (6)  13 (2)  58 (12)  51 (11) | 66 (46)  15 (11)  2 (1)  3 (2)  49 (35)  7 (5) | 117 (81)  13 (9)  2 (1)  0 (0)  9 (6)  4 (3) | r^2^=0.05, p<0.0001 |
| Role; n (%)  Nurse  Doctor  Allied Health Professional  Healthcare Assistant  Laboratory  Other | 556 (74)  76 (10)  30 (4)  65 (9)  10 (1)  18 (2) | 340 (73)  25 (5)  22 (5)  55 (12)  10 (2)  16 (3) | 103 (73)  24 (17)  8 (6)  6 (4)  0 (0)  1 (<1) | 113 (78)  27 (19)  0 (0)  4 (3)  0 (0)  1 (<1) | r^2^=0.03 p<0.0001 |

Positive IGRA results reported as percentage of total IGRAs performed, not of total cohort. ANOVA and Pearson’s Chi-squared test used, as appropriate.

*Supplemental Table 3: Characteristics of healthcare workers with positive interferon gamma release assays by site*

|  | Total (n=313) | Site 1 (n=112) | Site 2 (n=92) | Site 3 (n=15) | Site 4 (n=70) | Site 5 (n=24) | Statistics |
| --- | --- | --- | --- | --- | --- | --- | --- |
| Age, years; n (%)   - <30 - 30 – 39 - 40 – 49 - ≥50 | 54 (17)  186 (59)  57 (18)  16 (5) | 19 (17)  58 (52)  27 (24)  8 (7) | 14 (15)  55 (60)  18 (20)  5 (5) | 3 (2)  8 (50)  3 (19)  1 (6) | 13 (19)  52 (74)  4 (6)  1(1) | 4 (16)  13 (54)  6 (24)  1 (4) | r^2^=0.02, p=0.06 |
| Sex, female; n (%) | 229 (69) | 83 (74) | 60 (65) | 16 (100) | 47 (67) | 23 (92) | Χ^2^=14.93, p=0.005 |
| Staff role; n (%)   - Nurse - Doctor - Allied health - HCA - Laboratory - Other | 225 (72)  34 (11)  3 (<1)  31 (10)  5 (922)  15 (5) | 72 (64)  10 (9)  0 (0)  22 (20)  4 (4)  4 (4) | 60 (65)  15 (16)  3 (3)  3 (3)  1 (1)  10 (11) | 10 (67)  2 (13)  0 (0)  2 (13)  1 (6)  0 (0) | 63 (90)  4 (6)  0 (0)  3 (4)  0 (0)  0 (0) | 20 (83)  3 (13)  0 (0)  1 (4)  0 (0)  0 (0) | Χ^2^=52.59, p<0.0001 |
| Region of Birth; n (%)   - South & Central Asian - Sub-Saharan Africa - Eastern Europe - South American - East Asian - Other | 159 (51)  45 (15)  7 (2)  3 (<1)  84 (27)  15 (5) | 56 (50)  29 (26)  5 (4)  1 (17)  19 (2)  2 (2) | 63 (68)  11 (12)  0 (0)  2 (2)  10 (11)  6 (7) | 10 (69)  0 (0)  1 (6)  0 (0)  3 (19)  1 (6) | 12 (17)  2 (3)  1 (1)  0 (0)  50 (71)  5 (7) | 18 (75)  3 (13)  0 (0)  0 (0)  2 (8)  1 (4) | Χ^2^=123.87 p<0.0001 |
| Country of birth  South & Central Asian   - India - Pakistan - Saudi Arabia - Other   Sub-Saharan Africa   - South Africa - Ghana - Nigeria - Sudan - Zimbabwe - Other   Eastern Europe   - Poland - Romania   South American   - Brazil (3)   East Asian   - China - Philippines - Mongolia - Other   Other   - Ireland - Other | 121 (39)  26 (8)  4 (1)  8 (3)  11 (4)  2 (<1)  11 (4)  2 (<1)  11 (4)  9 (3)  4 (1)  2 (<1)  3 (<1)  8 (3)  72 (23)  2 (<1)  2 (<1)  10 (3)  5 (2) |  |  |  |  |  |  |

ANOVA (F score, r2) and Pearson’s Chi-squared test (Χ2) used, as appropriate. HCA=Healthcare attendant. Allied Health Professional is a composite of physiotherapists, occupational therapists, social workers, clinical nutritionists and speech and language therapists.

**Supplemental Table 4: Latent tuberculosis infection treatment characteristics across sites**

|  | Total (n=313) | Site 1 (n=112) | Site 2 (n=92) | Site 3 (n=15) | Site 4 (n=70) | Site 5 (n=24) | Statistics |
| --- | --- | --- | --- | --- | --- | --- | --- |
| Started treatment, yes; n (%) | 154 (49) | 30 (27) | 64 (70) | 14 (88) | 32 (46) | 14 (56) | Χ^2^=46.62, p<0.0001 |
| If not, reason why; n (%)   - Declined - DNA - Defer - Prior TB/LTBI - Other | 84 (27)  33 (11)  8 (3)  17 (5)  17 (5) | 47 (43)  18 (16)  5 (5)  6 (5)  4 (4) | 13 (14)  6 (7)  1 (1)  0 (0)  7 (8) | 0 (0)  1 (6)  1 (6)  0 (0)  0 (0) | 14 (20)  8 (11)  0 (0)  9 (13)  6 (9) | 9 (36)  0 (0)  1 (4)  1 (4)  0 (0) | Χ^2^=40.08, p=0.001 |
|  | Total  (n=154) | Site 1 (n=30) | Site 2 (n=64) | Site 3 (n=14) | Site 4 (n=32) | Site 5  (n=14) |  |
| Treatment choice; n (%)   - Rifampicin - Isoniazid | 140 (91)  14 (9) | 29 (97)  1 (3) | 59 (92)  5 (8) | 14 (100)  0 (0) | 32 (100)  0 (0) | 6 (43)  8 (57) | Χ^2^=45.04 p<0.0001 |
| Treatment completed, yes; n (%) | 127 (82) | 22 (73) | 52 (81) | 13 (93) | 28 (88) | 12 (86) | Χ^2^=3.50 p=0.48 |
| If not, why; n (%)   - DNA - Adverse effect | 14 (11)  13 (8) | 4 (13)  4 (13) | 8 (12)  4 (6) | 0 (0)  1 (6) | 1 (3)  3 (9) | 1 (7)  1 (7) | Χ^2^=3.30, p=0.51 |

Rates of treatment initiation, reasons for not commencing treatment, and treatment choice and outcomes. Pearson’s Chi-squared test used to assess differences. DNA=did not attend initial appointment for treatment consideration
